# Multifunctional luminescent nanomaterials from NaLa(MoO_4_)_2_:Eu^3+^/Tb^3+^ with tunable decay lifetimes, emission colors, and enhanced cell viability

**DOI:** 10.1038/srep11844

**Published:** 2015-08-11

**Authors:** Mei Yang, Youlong Liang, Qingyuan Gui, Bingxin Zhao, Dayong Jin, Mimi Lin, Lu Yan, Hongpeng You, Liming Dai, Yong Liu

**Affiliations:** 1 Institute of Advanced Materials for Nano-Bio Applications, School of Ophthalmology & Optometry, Wenzhou Medical University, Wenzhou, Zhejiang 325027, China; 2 Institute for Biomedical Materials and Devices, Faculty of Science, University of Technology Sydney, NSW, 2007, Australia; 3 Advanced Cytometry Labs, ARC Center of Excellence for Nanoscale BioPhotonics, Macquarie University, Sydney, NSW 2109, Australia; 4 State key Laboratory of Rare Earth Resource utilization Changchun Institute of Applied Chemistry, Chinese Academy of Sciences, Changchun, Jilin 130022, China; 5 Center of Advanced Science and Engineering for Carbon (Case4Carbon), Department of Macromolecular Science and Engineering, Case Western Reserve University, Cleveland, Ohio 44106, United States

## Abstract

A facile, but effective, method has been developed for large-scale preparation of NaLa(MoO_4_)_2_ nanorods and microflowers co-doped with Eu^3+^ and Tb^3+^ ions (abbreviated as: NLM:Ln^3+^). The as-synthesized nanomaterials possess a pure tetragonal phase with variable morphologies from shuttle-like nanorods to microflowers by controlling the reaction temperature and the amount of ethylene glycol used. Consequently, the resulting nanomaterials exhibit superb luminescent emissions over the visible region from red through yellow to green by simply changing the relative doping ratios of Eu^3+^ to Tb^3+^ ions. Biocompatibility study indicates that the addition of NLM:Ln^3+^ nanomaterials can stimulate the growth of normal human retinal pigment epithelium (ARPE-19) cells. Therefore, the newly-developed NaLa(MoO_4_)_2_ nanomaterials hold potentials for a wide range of multifunctional applications, including bioimaging, security protection, optical display, optoelectronics for information storage, and cell stimulation.

It has been a big challenge to fabricate highly luminescent fluorophores for efficient biomedical imaging. The recent rapid development in nanomaterails and nanotechnology provides possibilities for efficient and high-resolution bioimaging based on highly luminescent nanomaterials. In this context, various luminescent nanomaterials, such as Si nanoparticles^1^, semiconducting quantum dots (QDs)^2^,^3^, carbon dots^4^, nanodiamonds^5^,^6^ and lanthanide-doped nanomaterials^7^,^8^,^9^, have been developed for diverse applications, including biological imaging^10^. In particular, lanthanide-doped nanomaterials have been regarded as unique luminescent nanomaterials because of their outstanding luminescence properties, arising from the intra 4f and 4f-5d electron transitions of different lanthanide ions. For certain specific biological imaging applications, such as the time-gated detection of biological samples with suppressed interfering of autofluorescence and scattering, lanthanide-doped materials offer unique advantages, including narrow emission bands, and long luminescence decay times in the range of milliseconds^11^,^12^,^13^. Compared with other widely used luminescent nanomaterials (*e.g*., QDs), lanthanide-doped nanomaterials exhibit relatively lower biotoxicity and tunable emitting properties induced by changing the dopant elements in addition to the variation of nanoparticle sizes^14^.

Lanthanide molybdates, a typical class of double molybdates, have attracted a great deal of attention recently due to their technological potentials for multifunctional applications, such as ferroelectricity and ferroelasticity, ionic conducting of oxygen, lasing, and phosphors luminescense^15^,^16^,^17^. The general formula of double molybdates is MLn(MoO_4_)_2_, where M is a monovalent alkalication (Li–Cs). Among them, NaLn(MoO_4_)_2_ is of particular interest due to its wide applications as crystalline laser materials using the Czochralski method^18^. However, the potential use of double molybdates beyond the crystalline laser materials has rarely been discussed. Recent progress in controllable synthesis of rear earth molybdates with specific morphologies and sizes provides possible ways to broaden their applications^15^,^16^. There is an increasing demand of lanthanide-based fluorescent nanomaterials with multiple emitting colors for some specific applications such as light display systems, lasers, and optoelectronic devices^19^,^20^,^21^. To realize the multi-color emissions, a facile and efficient strategy to be considered is to change the relative content of activators rather than to vary the excitaion center. In this work, for instance, we have realized a wide range of emission colors over red, orange, yellow, green-yellow, and green by simply changing the relative doping concentrations of Eu^3+^ and Tb^3+^ ions during the preparation of NaLa(MoO_4_)_2_:Ln^3+^ nanorods (microflowers). Generally speaking, the illuminescence lifetime will not be affected by initial perturbation conditions, such as wavelength of excitation, duration of light exposure, one- or multiphoton excitation and photobleaching^22^. Tunable luminescence lifetime of lathanide-based nanomaterials, howerver, has been achieved, via the control of nanomaterial size^23^ and the sensitizer to activator concentration ratio^24^, for various applications, indcluding multiplexed biological encoding, security proetction, and data storage^25^,^26^.

On the other hand, it is well known that physiochemical properties of nanomaterials can be influenced by their sizes, shapes, surface features, and crystal structures. Morphology and/or size controllable preparation is essential for property tuning, including fluorescent properties of lanthanide-based materials^27^. Herein, we report our recent findings on the controlled synthesis of NaLa(MoO_4_)_2_:Ln^3+^(Ln^3+^ = Eu^3+^,Tb^3+^) (abbreviated as: NLM:Ln^3+^) nanomaterials with controllable morphologies, from nanorods to microflowers of various sizes in the range of 10 nm to 3 μm, and tunable fluorescence properties. In the present work, we have developed a simply, but efficient, way to tune luminescence lifetime of NaLa(MoO_4_)_2_:Ln^3+^ nanorods (microflowers) by simply adjusting the relative co-doping ratio of Eu^3+^ to Tb^3+^. We have also systematically studied the luminescent and biocompatible properties of the resulting nanomaterials. The as-synthesized nanomaterials are demonstrated to exhibit superb luminescent properties with tunable decay lifetimes and emission colors over the range of red, orange, yellow, green-yellow, and green by simply changing the relative doping concentrations of Eu^3+^ and Tb^3+^ ions. This work suggests potential applications in decoding of multiplexing in life science, which requires simultaneous identification of multiple species^28^ and other applications beyond crystalline laser materials, such as multichannel bioimaging, high-density data storage, and security protection.

## Results

**Figure 1:**
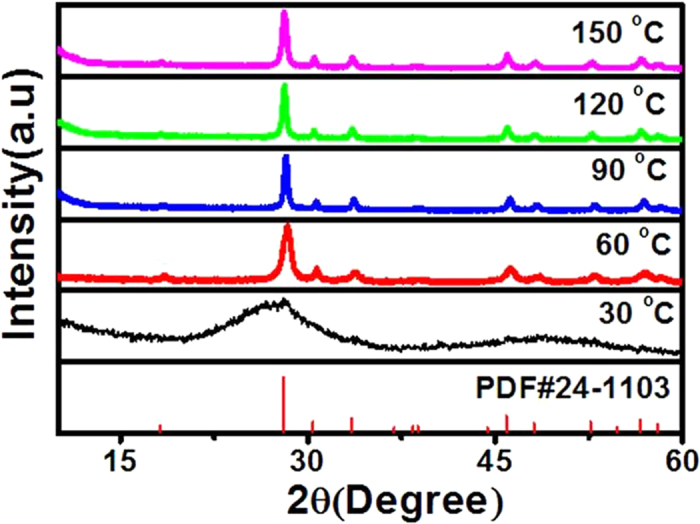
XRD patterns. XRD patterns of the samples obtained at different temperatures for 10 h and the standard PDF card of tetragonal phase NLM:Ln^3+^(Ln^3+^ = Eu^3+^,Tb^3+^).

**Figure 2:**
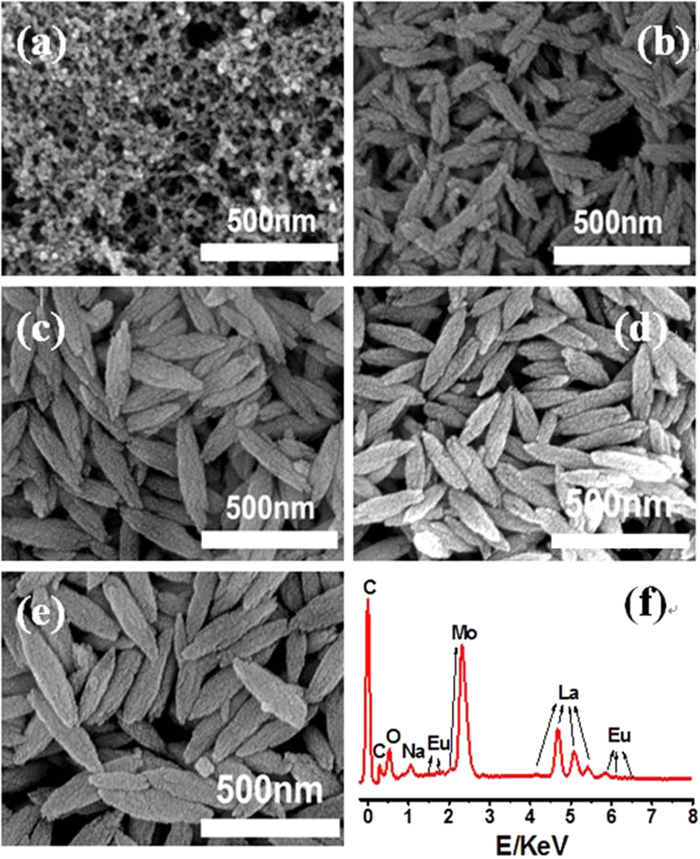
SEM micrographs and EDX spectrum. **a**, SEM image of the NLM:Ln^3+^ nanomaterials obtained at 30 ^o^C for 10 h. **b,** SEM image of the NLM:Ln^3+^ nanomaterials obtained at 60 ^o^C for 10 h. **c,** SEM image of the NLM:Ln^3+^ nanomaterials obtained at 90 ^o^C for 10 h. **d**, SEM image of the NLM:Ln^3+^ nanomaterials obtained at 120 ^o^C for 10 h. **e**, SEM image of the NLM:Ln^3+^ nanomaterials obtained at150 ^o^C for 10 h. **f,** EDX spectrum of the NLM:Ln^3+^ nanomaterials prepared at 90 ^o^C for 10 h.

Figure 1 shows the XRD patterns of the resulting NLM:Ln^3+^ nanomaterials obtained at different temperatures.The peak positions of all XRD patterns shown in Fig. 1 fitted well with those of the tetragonal phase NaLa(MoO_4_)_2_ in JCPDS file 24–1103. No additional peak of other phases had been found for all samples investigated in this study. As seen in Fig. 1, NLM:Ln^3+^ nanomaterials with the pure tetragonal phase could be obtained in a broad temperature range from 30 to 150 °C. Below 90 °C, the XRD peak intensities increased gradually with increasing the reaction temperature, indicating a thermally enhanced crystalization. Above 90 °C , however, the diffraction peak intensities remained unchanged. XRD patterns of the NaLa(MoO_4_)_2_:5% Eu^3+^, NaLa(MoO_4_)_2_:5% Tb^3+^, and NaLa(MoO_4_)_2_:2% Eu^3+^, 3% Tb^3+^ are shown in Figure S1 (Supplementary Information, SI). All samples show similar XRD patterns as the pure NaLa(MoO_4_)_2,_ suggesting that there is no phase change occurred during doping of Eu^3+^ and Tb^3+^. This may be attributed to the small amounts of doing ions, and similar properties and ionic radius between the doping ions and La. The corresponding SEM images given in Fig. 2 indicate that the reaction temperature plays an important role in regulating morphologies of the resultant NaLa(MoO_4_)_2_ samples. As shown in Fig. 2a, well-interconnected nanoparticles were obtained for the sample prepared at 30 °C. Upon increasing the synthesis temperature up to 60 °C, single shuttle-like nanorods composed of nanoparticles with about 200 nm in length and 30 nm in diameter at the middle part were produced (Fig. 2b). Further temperature increased to 90 °C or above (120 °C, 150 °C) caused almost no more morphology change except the slightly increased size (Fig. 2c–e). These results suggest that the reaction temperature has important effects on the morphology and size of the resulting NaLa(MoO_4_)_2_ nanomaterials. The energy dispersive X-ray spectrum (EDX) of the as-synthesized shuttle-like nanorods (Fig. 2f) further confirms the presence of La, Eu, Na, Mo, and O in the resulting nanomaterials (C was from the substrate).

**Figure 3:**
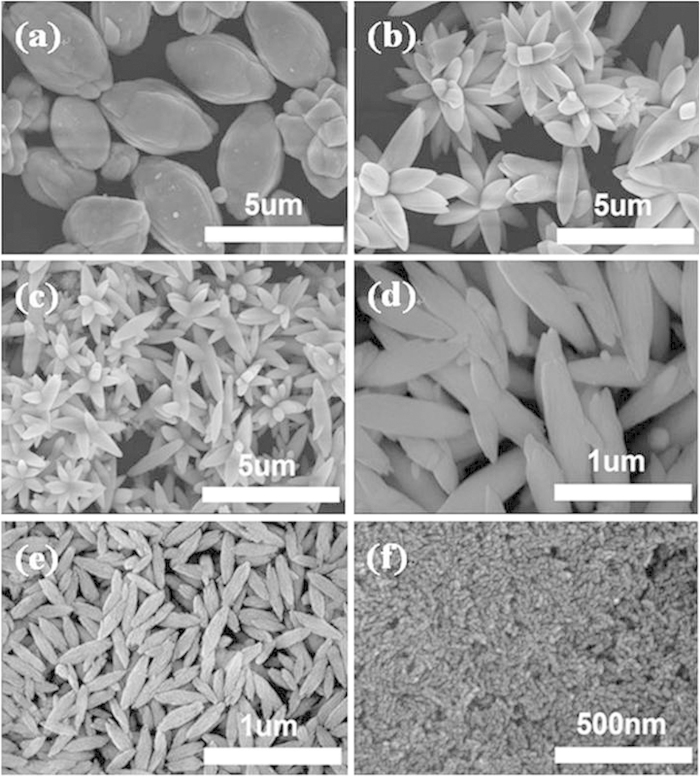
SEM micrographs of NLM:Ln^3+^ micro/nano- materials obtained at 90 ^o^C for 10 h with addition of various amounts of ethylene glycol. **a**, 0 mL. **b**, 10 mL. **c**, 20 mL. **d**, 25 mL. **e**, 30 mL. **f**, 40 mL.

Apart from the reaction temperature, the amount of ethylene glycol (EG) added in the reaction solution was found to be critical in determining the final morphology and size for the resultant NLM:Ln^3+^ nanomaterials. Without the addition of ethylene glycol into the reaction system, large shuttle-like microrods with a diameter of 3 μm (the middle part) and length of 5 μm formed (Fig. 3a). Beautiful flowerlike architectures composed of shuttle microrods with an average diameter of 1 μm and length of 3–5 μm were observed when 10 mL ethylene glycol was introduced (Fig. 3b). Continued addition of EG up to 20 mL caused a decreas in the size of the as-synthesized micro-flowers to 500 nm in diameter and 2–3 μm in length (Fig. 3c). Further increase in the content of EG to 25 mL, however, led to disappearance of the flower structure and a concomitant recovery of the shuttle-like rods with a further reduced diameter of 200 nm and 1 μm in length (Fig. 3d). Nanoscaled shuttle-like rods with 60 nm in diameter (the middle part) and 300 nm in length were obtained when the amount of EG was further increased to 30 mL (Fig. 3e). The average size of the nanorods could be further reduced to 10 nm in diameter and 100 nm in length by introduction of 40 mL EG (Fig. 3f). These results indicate that both the morphology and size of the NLM:Ln^3+^ nanomaterials could be regulated by controlling the amount of EG to be added into the reaction solutions. Our XRD measurements on the resulting NLM:Ln^3+^ nanomaterials (Figure S2, SI) revealed that the peak positions of all XRD patterns agreed well with those of the tetragonal phase NaLa(MoO_4_)_2_ in JCPDS file 24–1103. No additional peak of other phases had been found in all the samples studied, indicating that the addition of EG with amounts covered by this work had little effect on the phase structure of the resulting materials.

**Figure 4:**
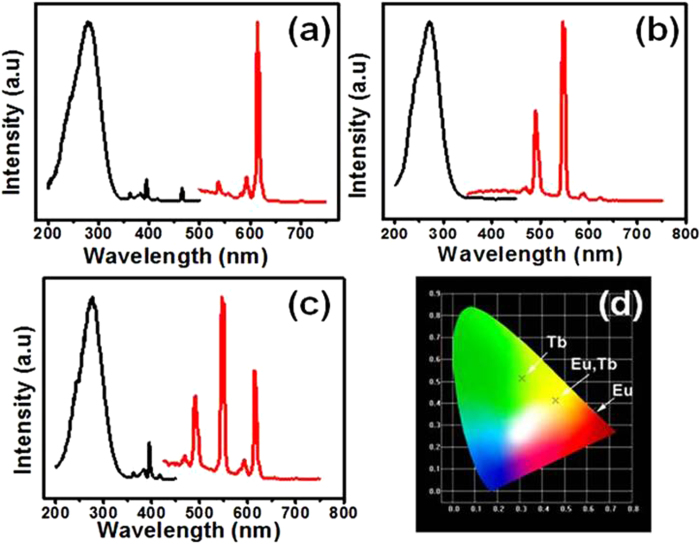
Fluorescence performance. **a,** The excitation and emission spectra of NLM:Eu^3+^. **b,** The excitation and emission spectra of NLM:Tb^3+^. **c,** The excitation and emission spectra of NLM:2% Eu^3+^/3% Tb^3+^. **d,** CIE chromaticity diagram for the emission spectra of the above three samples.

Luminescent properties of the shuttle-like NLM:Ln^3+^ nanorods doped with different lanthanide ions were investigated (Fig. 4). Fig. 4a displays the excitation and emission spectra of NLM:Eu^3+^ nanorods. The excitation spectrum was obtained by monitoring at 615 nm, which consisted of a strong broadband and several weak peaks. The broadband ranging from 200 to 350 nm centered at 278 nm is associated with the O–Mo charge transfer (CT) transition. Weak peaks in the longer wavelength region (360–500 nm) are assigned to the general f-f transitions within the Eu^3+^ 4f^6^ electron configuration. The weak bands at 395 and 466 nm are associated with the ^7^F_0_→^5^L_6_ and ^7^F_0_→^5^D_2_ transitions of the Eu^3+^ ions. Upon excited at 278 nm, the NLM:Eu^3+^ nanorods exhibited a strong red luminescence. The emission spectrum consists of the ^5^D_0_→^7^F_J_ (J = 1, 2) emission lines of the Eu^3+^ ions with a strong peak at 616 nm and a weak peak at 594 nm. As a symmetry parameter of the coordinated polyhedron around the Eu^3+^ ions, the asymmetry ratio R (R = I (^5^D_0_ →^7^F_2_)/I(^5^D_0_→^7^F_1_)) has been widely used to measure the symmetry of the crystal site. In this NaLa(MoO_4_)_2_ tetragonal phase, the high R indicates that Eu^3+^ occupies a center of asymmetry, which is useful for improvement of the color purity for the red phosphor. Fig. 4b shows the excitation and emission spectra of NLM:Tb^3+^ nanorods. The excitation spectrum monitored with the ^5^D_4_→^7^F_5_ transition at 544 nm composes of a strong and broad band from 200 to 350 nm centered at 274 nm, corresponding to the charge-transfer transitions within the MoO_4_^2-^ groups. Four bands of f-f transition lines within 4f^8^ electron configuration of Tb^3+^ can be observed from the emission spectrum excited at 274 nm, including ^5^D_4_→^7^F_6_ (490 nm) in the blue region, ^5^D_4_→^7^F_5_ (546 nm, strongest) in the green region, ^5^D_4_→^7^F_4_ (587 nm) and ^5^D_4_→^7^F_3_ (621 nm) in the red region. These results suggest that an efficient energy transfer from MoO_4_^2−^ to Tb^3+^ has occurred. Yellow light can be further observed when we co-doped Eu^3+^ and Tb^3+^ ions into the NLM system and excited at 280 nm. As shown in Fig. 4c, the excitation and emission characteristic peaks of Eu^3+^ and Tb^3+^ ions were observed simultaneously. The results from the above-mentioned samples were further confirmed by the corresponding CIE (Commission Internationale de L'Éclairage, 1931) chromaticity diagram (Fig. 4d).

**Figure 5:**
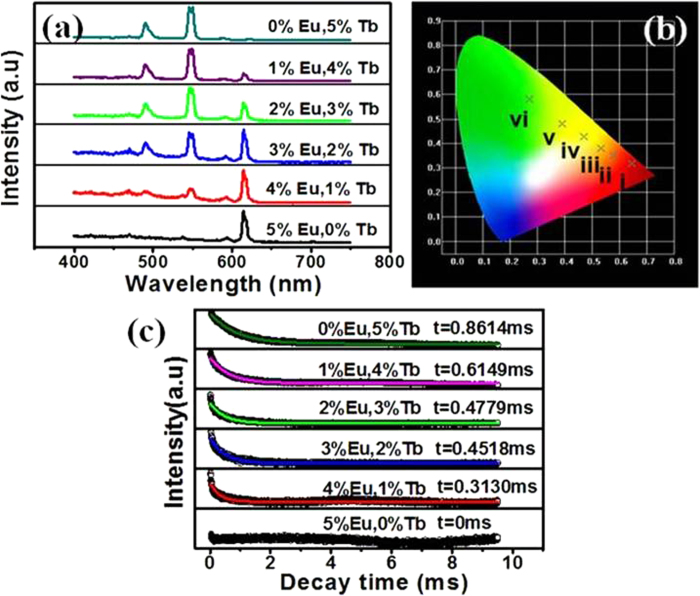
Changes of fluorescence performances with various ratios of Eu^3+^ to Tb^3+^. **a,** Photoluminescence emission spectra of the Eu^3+^ and Tb^3+^ co-doped NLM nanorods under the excitation at 280 nm (total concentration: 5 mol%). **b,** CIE chromaticity diagram for the emission spectra of the various ratios of Eu^3+^ to Tb^3+^ co-doped NLM nanorods (i: 5% Eu^3+^; ii: 4% Eu^3+^/1% Tb^3+^; iii: 3% Eu^3+^/2% Tb^3+^; iv: 2% Eu^3+^/3% Tb^3+^; v:- 1% Eu^3+^/4% Tb^3+^; vi: 5% Tb^3+^). **c,** the decay curves of Tb^3+^ in the resulting NLM nanorods. The sample was excited at 289 nm (monitored at 544 nm).

In order to investigate the possible tunable luminescent properties of the resulting NLM:Ln^3+^ nanorods in details, various amounts of Eu^3+^ and Tb^3+^ ions were doped into the NLM host lattice (the total concentration is 5 mol %). As shown in Fig. 5a, the as-prepared pure Eu^3+^ doped NLM showed strong red emission under UV light excitation. When Tb^3+^ ions were gradually co-doped into the NLM host lattice, the characteristic emission of Tb^3+^ ions was observed besides the Eu^3+^ emission. With increasing the relative concentration ratios of Tb^3+^ to Eu^3+^, the luminescence intensity of Eu^3+^ ions gradually decreased while the emission of Tb^3+^ increased significantly (Fig. 5a). Furthermore, pure Tb^3+^ doped NLM without addition of Eu^3+^ showed bright green emission. Accordingly, the photoluminescence colors were thus tunable over the wide range of the red region to the green region by simply adjusting the relative doping concentration ratios of Tb^3+^ to Eu^3+^ ions. The results were further confirmed by the corresponding CIE (Fig. 5b). These results indicate that the as-synthesized NLM:Ln^3+^ nanorod phosphors show multicolor emissions in the visible region when excited by a single wavelength light, providing promising possibilities for various potential applications, such as bioimaging, optical display, and optoelectronics.

We also found that the dopant concentration of the emission ions had an influence on the lifetime of luminescence. As shown in Fig. 5c, the decay curves of Tb^3+^ were well fitted into single-exponential function as I = I_0_ exp-(- t/τ) (where τ is the 1/e lifetime of the Tb^3+^ ions). The lifetimes of Tb^3+^ were determined to be 0 (for 5% Eu^3+^ sole doping), 0.3130 (for 1% Tb^3+^/4%Eu^3+^), 0.4518 (for 2% Tb^3+^/^3%^Eu^3+^), 0.4779 (for 3% Tb^3+^/2%Eu^3+^), 0.6149 (4% Tb^3+^/1%Eu^3+)^ and 0.8614 ms (for 5%Tb^3+^), respectively. The lifetime increased gradually with increasing ratios of Tb^3+^ to Eu^3+^. Clearly, therefore, Figs 4,5 indicate that tunable luminescent life times with various colors and intensities are achieved by varying co-doping ratios of Tb^3+^ to Eu^3+^ in NLM nanomaterials, which can be potentially used for decoding multiplexing along with many other applications^25^.

We further studied influence of the reaction temperature and the EG amount on the luminescent properties of the resulting NLM:Ln^3+^ nanomaterials. It ws noted that the luminescent intensity increased gradually with increasing the reaction temperature up to 120 ^o^C (Figure S3, SI), probably attributable to the improved perfection of the crystal phase structures and/or their increased sizes with increasing reaction temperature as discussed in Figs 1,2. Further increase in the reaction temperature up to 150 ^o^C, however, caused a slight decrease in the luminescence intensity of the resulting material, presumabaly because the sample has changed its color to grey at 150 ^o^C. As seen in Figure S4 (SI), the luminescent intensity could also be enhanced by decreasing the amount of EG since the reduced content of EG could cause an increase in the size of the resulting material (Fig. 3). As is well-known, the luminescence intensity of rare earth nanomaterials generally depends on the radiative relaxation from a higher excited state to a ground state, and hence the surface characteristics, such as atomic arrangement, composition, surface topography, surface defect, and adsorbed gas, are essential for nonradiative recombination and quenching of the luminescent mechanisms. Thus, changes in surface morphologies and properties greatly influence the emission intensity of the nanophosphors by nonradiative mechanisms in luminescence processes^29^.

## Discussion

**Figure 6:**
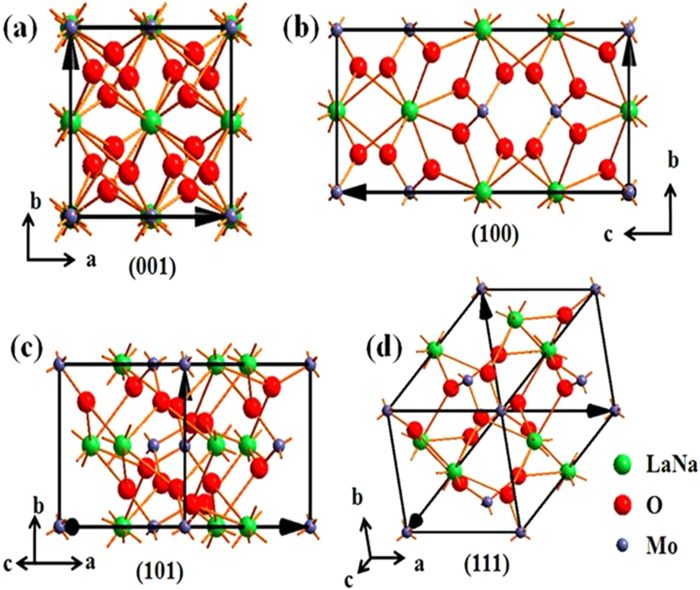
Simulated crystal structure. Simulated crystal structures of tetragonal NaLa(MoO_4_)_2_with different crystal faces.

To gain a better understanding of the growth mechanism of the NaLa(MoO_4_)_2_:Ln^3+^ nanostructure, we have investigated several factors influencing the growth of nanomaterials, including the reaction time (Figure S5 and S6), the reaction temperature (Figure S7), and the EG amount introduced into the reaction solution. As well known, it is the relative growth rate of different crystal facets that gives rise to the various morphologies of the resulting materials^30^. As shown in Fig. 6, the simulated crystal structures of the tetragonal NaLa(MoO_4_)_2_ nanomaterials with different crystal facets show a relatively higher packing density of Na^+^ and La^3+^ ions on the (001) and (101) planes (Fig. 6a,c) than those on the other facets (Fig. 6b,d). Upon introduction of EG, EG molecules may prefer to absorb on the (001) and (101) facets, via ligand bonds with the La^3+^ ions, to hinder the crystal growth along the (001) and (101) directions whilst promoting the growth along the (100) and the other equivalent direction, leading to the formation of the observed shuttle-like nanorods. In addition, surface energy is considered as another essential factor for formation of the resulting structure^31^. Reduction in surface energy facilitates the growth of simple particles. The surface energy of the tetragonal NaLa(MoO_4_)_2_ on the (100)crystal facet is higher than that on the (111) facet, leading to the faster growth along the (100) direction than the other direction and the subsequently disappearance of this crystal facet^32^, resulting in the formation of shuttle-like crystals. Furthermore, lower disappearing rate of the (001) facet facilitates the formation of the shuttle-like nanorods with longer axis.

**Figure 7:**
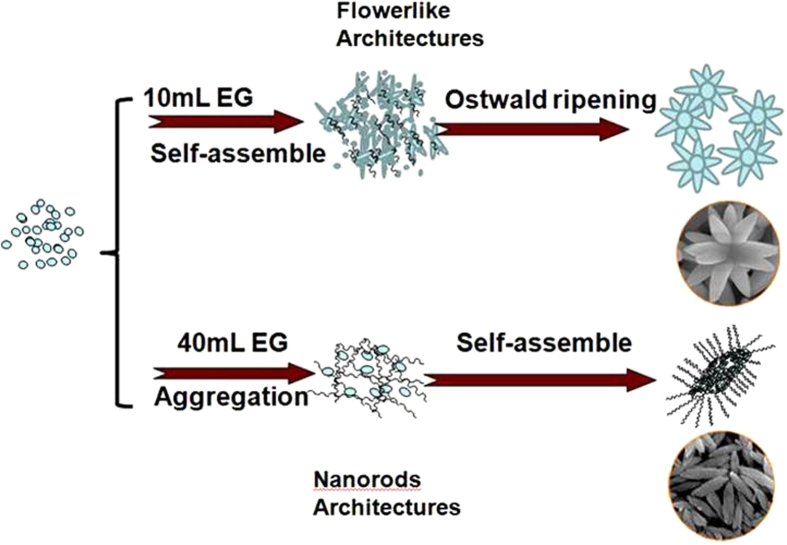
Schematic formation of different mophorlogies. Schematic illustration of the morphology evolution from shuttle-like nanorods to flower-like architectures.

The presence of EG plays another important role in determining the final morphology by affecting the nuclei aggregation through ligand reactions between the two –OH functional groups of EG and lanthanide ions. The crystal tends to be the large shuttle-like microrods when there is no EG introduced. Upon the introduction of a small amount of EG, microflowers composed of micro/nanorods formed (Fig. 3b,c) under co-effects of initial nuclei aggregation and crystal growth, as schematically shown in Fig. 7. The newly-formed microflowers then underwent the Ostwald ripening process with consumption of small nanoparticles. Further increase in the amount of EG led to the formation of nanorods composed of nanoparticles (Fig. 3e,f), due presumably to the increased viscosity of solution that promoted the formation of large amounts of nuclei while further growth of the nuclei is restricted by the limited content of raw materials. Moreover, the presence of EG caused a decrease in the surface energy, leading to the formation of nanorods from nanoparticles (Fig. 7). It is thus concluded that formation of nanorods and microflowers are arisen from the co-effects of the crystal structure of tetragonal NaLa(MoO_4_)_2_ and the presence of EG.

In this work, we further found that the luminescent color and the emission lifetime of the resulting NaLa(MoO_4_)_2_:Ln^3+^ were tunable by controlling the relative co-doping ratios of Tb^3+^ to Eu^3+^. The luminescence colors were tunable over a wide range from the red region to green region due to adjustable emission peaks from Tb^3+^ and Eu^3+^ and energy transfer between Tb^3+^ and Eu^3+^^33^. The tunable luminescence decay time with different relative amount ratios of Tb^3+^ to Eu^3+^ can be attributed to the increased amount of Tb^3+^ (from 0, 1%, 2%, 3%, 4% to 5% in the total concentration of 5%) which leads to the enhanced luminescence intensity beyond energy transfer between Tb^3+^ and Eu^3+^^34^,^35^. As shown in Fig. 5a, the increased Tb^3+^ amounts lead to the enhanced emission intensity of Tb^3+^ beyond energy transfer between Tb^3+^ and Eu^3+^, resulting in the increased luminescence decay time.

**Figure 8:**
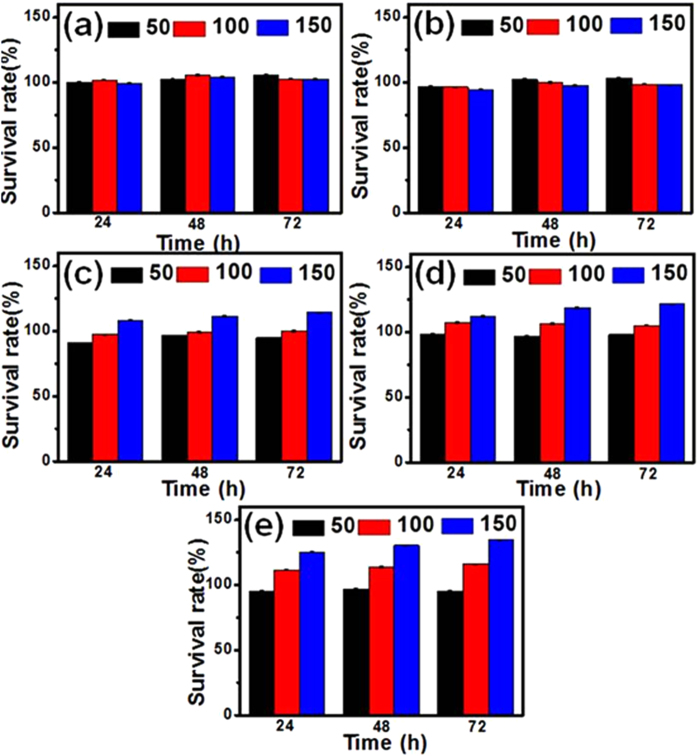
Cell viability of ARPE-19 cells incubated with NLM:Ln^3+^ materials with different morphologies. **a,** Microrods shown in Figure 3a. **b,** Micro-flowers in Fig. 3b. **c,** Micro-flowers in Fig. 3c. **d,** Nanorods in Fig. 3e. **e,** Nanorods in Fig. 3f.

For the purpose of *in vivo* bio-applications, it is essential to evaluate biocompatibility of the resulting nanomaterials. *In vitro* cytotoxicity of the as-synthesized NLM:Ln^3+^ nanomaterials with different morphologies and sizes was thus measured using the ARPE-19 cell which is a cell line derived from human retina pigment epithelium (RPE) cells. As shown in Fig. 8, all samples showed an excellent biocompatibility with ARPE-19 cells even after a prelonged incubation time (72 h). Particularly, we found that the size of the nanomaterials significantly influenced the cell viability. When the size of the resulting NLM:Ln^3+^ was at the microscale, increasing concentrations of nanomaterials didn’t cause changes in cell viability (Fig. 8a,b). When the size of the nanomaterials was decreased to the nanoscale, however, the cell viability increased significantly with increasing nanomaterial concentrations (Fig. 8d,8e). The improved cell viability may be due to the enhanced surface area of the samples when the size is decreased to the nanoscale, which provides more suitable environment for the cell adhesion and growth. These *in-vitro* cytotoxicity results suggest a low toxicity for the NaLa(MoO_4_)_2_:Ln^3+^ materials while the growth of cells can even be stimulated by increasing concentrations of nanoscaled NaLa(MoO_4_)_2_:Ln^3+^.

## Conclusions

We have presented a facile, but effective, approach for preparation of Eu^3+^ and Tb^3+^ co-doped NLM:Ln^3+^ nanomaterials with tunable shapes (e.g., shuttle-like micronanorods, micro-flowers, shuttle-like nanorods). XRD patterns confirmed that the as-synthesized nanomaterials were pure tetragonal phase. SEM images revealed that various morphologies of the resulting nanomaterials could be realized by adjusting the reaction temperatures and the amounts of ethylene glycol introduced. The Eu^3+^ and Tb^3+^ co-doped NLM:Ln^3+^ nanorods exhibited excellent luminescent properties with tunable life times, and luminescent colors in a wide range of emission region, including red, orange, yellow, green-yellow, and green, by varying the relative doping ratios of Tb^3+^ to Eu^3+^. These results suggest their potential applications in diverse fields, such as multichannel bioimaging, high-density data storage, security protection, optical display, and optoelectronics. *In vitro* cytotoxicity study indicates that the resulting nanomaterials are highly biocompitable and can stimulate the growth of human normal cells. Our preliminary results show that the novel biocompatible Eu^3+^/Tb^3+^ co-doped NLM:Ln^3+^ nanomaterials exhibit unique morphologies and sizes with multifunctional tunable properties, including tunable luminescent colors, intensities, and lifetimes, opening opportunities for the development of novel multifunctional hybrid nanomaterials for a large variety of applications ranging from nanomedicine to our daily life science.

## Methods

### Materials and Reagents

La_2_O_3_ (99.999%), Eu_2_O_3_ (99.999%), Tb_4_O_7_ (99.999%) were purchased from Wuxi Yiteng Rare-Earth Limited Corporation. All the other analytical grade reagents were used as received without further purification.

### NaLa(MoO_4_)_2_:Ln^3+^ (Ln^3+^ = Eu^3+^/Tb^3+^) Synthesis

La(NO_3_)_3_·6H_2_O, Eu(NO_3_)_3_·6H_2_O, and Tb(NO_3_)_3_·6H_2_O were obtained by dissolving La_2_O_3_, Eu_2_O_3_ and Tb_4_O_7_ in dilute HNO_3_ solution under heating with agitation separately, and followed by evaporation until desired products were obtained.

In a typical synthesis, 1 mmol La(NO_3_)_3_·6H_2_O was dissolved in ethylene glycol (EG). 2 mmol Na_2_MoO_4_·2H_2_O aqueous solution was then added under magnetic stirring. The mixed solution was transferred into a 100 mL round flask, which was maintained at 30–90 °C for 10 h without refluxing. After cooling to the room temperature naturally, the precipitate was collected and washed with deionized water and ethanol for several times. Then, the final products were dried at 60 °C for 24 h in air.

A similar process was employed to prepare Eu^3+^ (5 mol %), Tb^3+^ (5 mol %) doped NLM samples with addition of Eu(NO_3_)_3_ and Tb(NO_3_)_3_ into La(NO_3_)_3_ aqueous solution at the first step.

### Cell Culture

*In vitro* cytotoxicity measurements were performed as follows: 5 × 10^3^/well ARPE-19 cells were seeded into 96-well plate and 24 h later for the cell adherence. The five kinds of materials with different morphologies and sizes were added with the concentration of 50, 100, and 150 μg/ml, respectively. The number of every group with samples was more than 6. The time of the cell incubated was controlled for 24, 48 and 72 h. The cell viability was detected by CCK-8 test and the experiment was repeated for three times.

### Characterization

Morphologies of the resulting materials were examined on a field emission scanning electron microscope (FESEM) (HITACHI S-4800). XRD patterns were measured on a Rigaku-D X-ray powder Diffractometer with Cu K radiation (λ = 1.54 Å). Excitation and emission spectra were recorded by a Hitachi F-4500 fluorescence spectrophotometer equipped with a 150 W xenon lamp as the excitation source. The luminescence decay curves were carried out with a Lecroy Wave Runner 6100 digital oscilloscope (1 GHz) using a tunable laser (pulse width = 4 ns, gate = 50 ns) as the excitation source (Continuum Sunlite OPO). CCK-8 test was operated at SpectraMax M5 Molecular Devices. All measurements were performed at the room temperature unless otherwise stated.

## Additional Information

**How to cite this article**: Yang, M. *et al.* Multifunctional luminescent nanomaterials from NaLa(MoO_4_)_2_:Eu^3+^/Tb^3+^ with tunable decay lifetimes, emission colors, and enhanced cell viability. *Sci. Rep.* **5**, 11844; doi: 10.1038/srep11844 (2015).

## Supplementary Material

Supplementary Information
