# Supplementary material for: Multifunctional luminescent nanomaterials from NaLa(MoO4)2:Eu3+/Tb3+ with tunable decay lifetimes, emission colors, and enhanced cell viability
Source: Sci Rep. 2015 Aug 11;5:11844. doi: 10.1038/srep11844 (PMC4531290; doi:10.1038/srep11844)
Supplement: Supplementary Information [file srep11844-s1.doc]

**Supplementary Information**

Multifunctional luminescent nanomaterials from NaLa(MoO_4_)_2_:Eu^3+^/Tb^3+^ with tunable decay lifetimes, emission colors, and enhanced cell viability

Mei Yang,^1,6^ Youlong Liang,^1,6^ Qingyuan Gui,^1^ Bingxin Zhao,^1^ Dayong Jin,^2,3^Mimi Lin,^1^ Lu Yan,^1^ Hongpeng You,^4^ Liming Dai,^1,5,*^, and Yong Liu^1,3,*^

*^1^Institute of Advanced Materials for Nano-Bio Applications, School of Ophthalmology & Optometry, Wenzhou Medical University, 270 Xueyuan Xi Road, Wenzhou, Zhejiang 325027, China*

*^2^Institute for Biomedical Materials and Devices, Faculty of Science, University of Technology Sydney, NSW, 2007, Australia.*

*^3^Advanced Cytometry Labs, ARC Center of Excellence for Nanoscale BioPhotonics, Macquarie University, Sydney, NSW 2109, Australia.*

*^4^State key Laboratory of Rare Earth Resource utilization Changchun Institute of Applied Chemistry, Chinese Academy of Sciences, Changchun, Jilin 130022, China.*

*^5^Center of Advanced Science and Engineering for Carbon (Case4Carbon), Department of Macromolecular Science and Engineering, Case Western Reserve University, Cleveland, Ohio 44106, United States.*

*^6^These authors contributed equally.*

*Correspondence should be addressed to Y. L. (yongliu1980@hotmail.com), and

L. D. (liming.dai@case.edu)


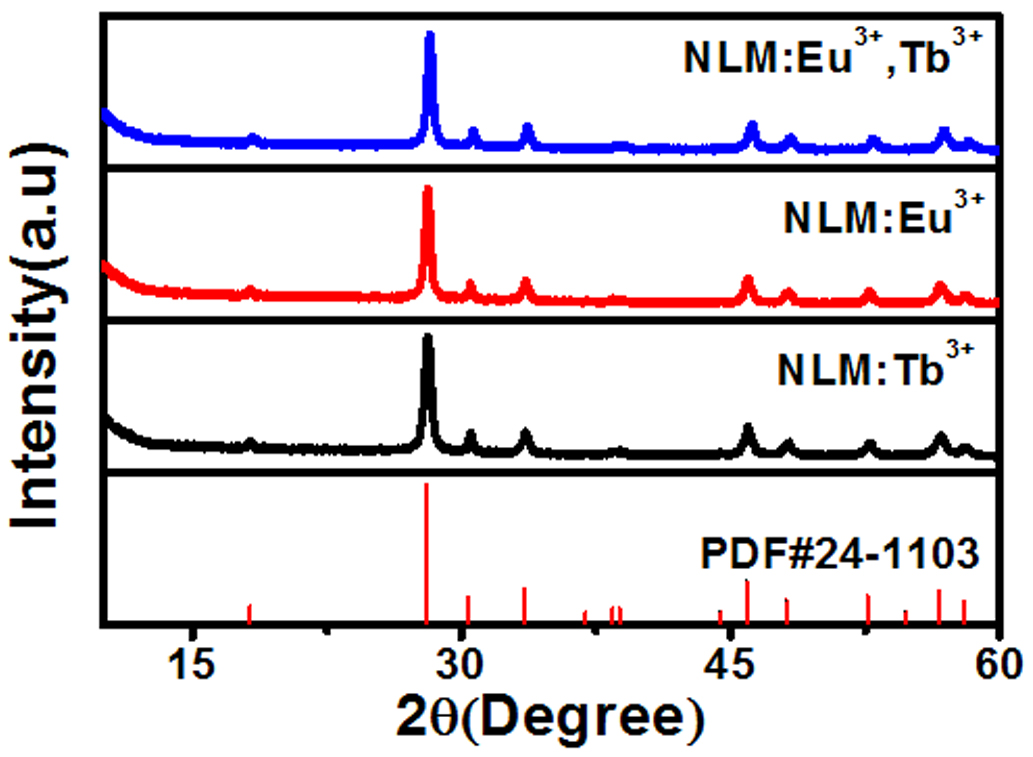


Figure S1. XRD patterns of the NLM:2% Eu^3+^, 3% Tb^3+^ (blue line), the NLM:5% Eu^3+^ (red line), and the NLM: 5% Tb^3+^ (black line) obtained at 90 °C over 10 h.


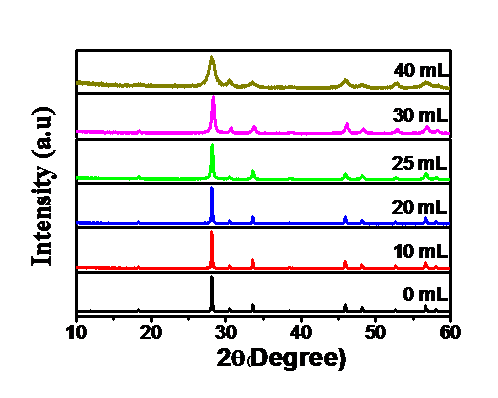


Figure S2. XRD patterns of the NLM:Ln^3+^ obtained at 90 °C over 10 h with different amounts of ethylene glycol.


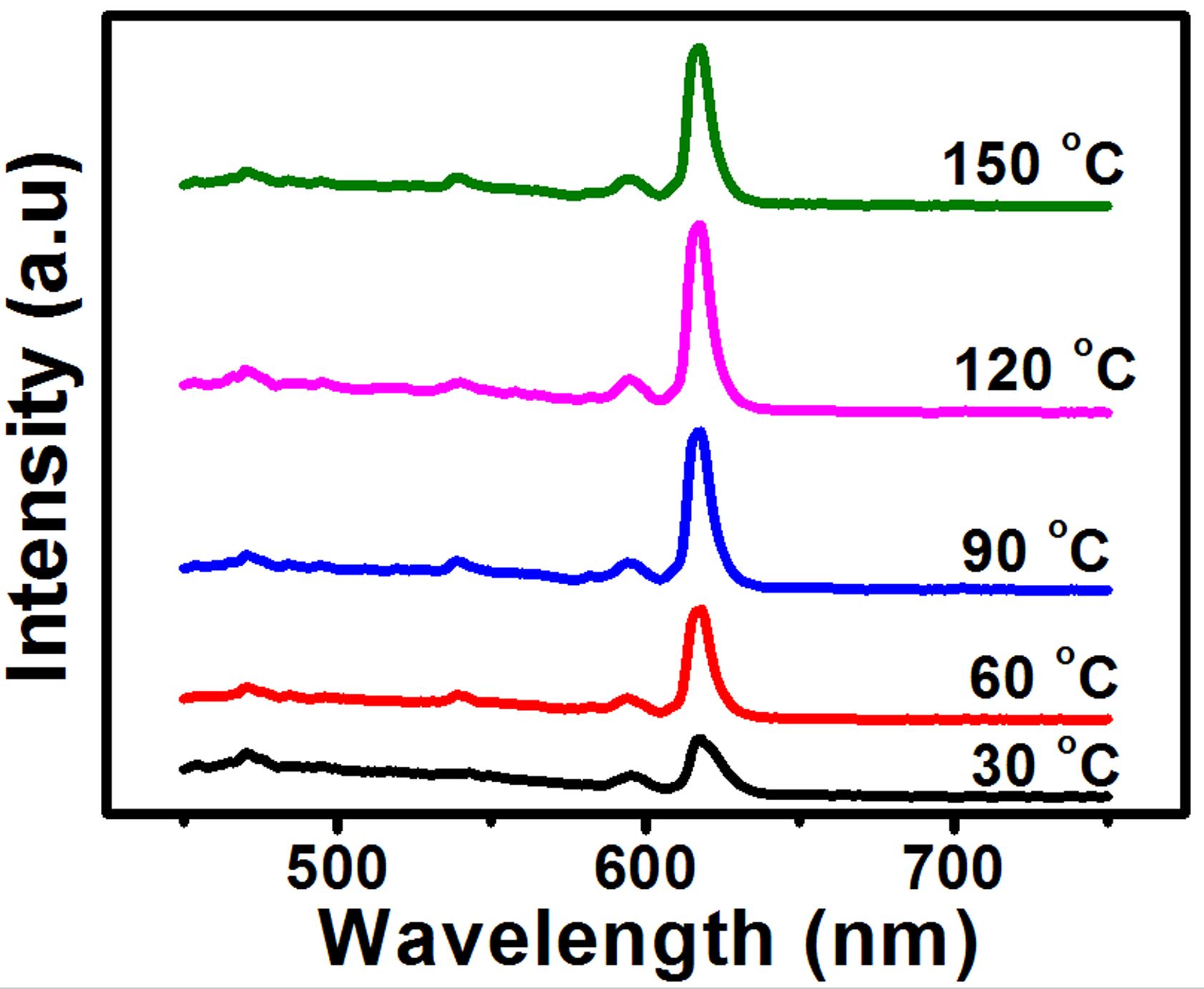


Figure S3. Emission spectra of the NLM:Ln^3+^ obtained at different temperature over 10 h with addition of 30 mL ethylene glycol.

Figure S4. Emission spectra of the NLM:Ln^3+^ obtained at 90 °C over 10 h with different amounts of ethylene glycol.

Influence of the reaction time on the morphology was investigated. When the EG amount was controlled at 30 mL, nanorods was observed. Nanoparticles were obtained at the initial step. Mixture of nanoparticles and nanorods were identified after the reaction for a half of an hour. Pure nanorodes could be obtained after reaction for 1 h. No further change in morphology was found after reaction for 10 h.


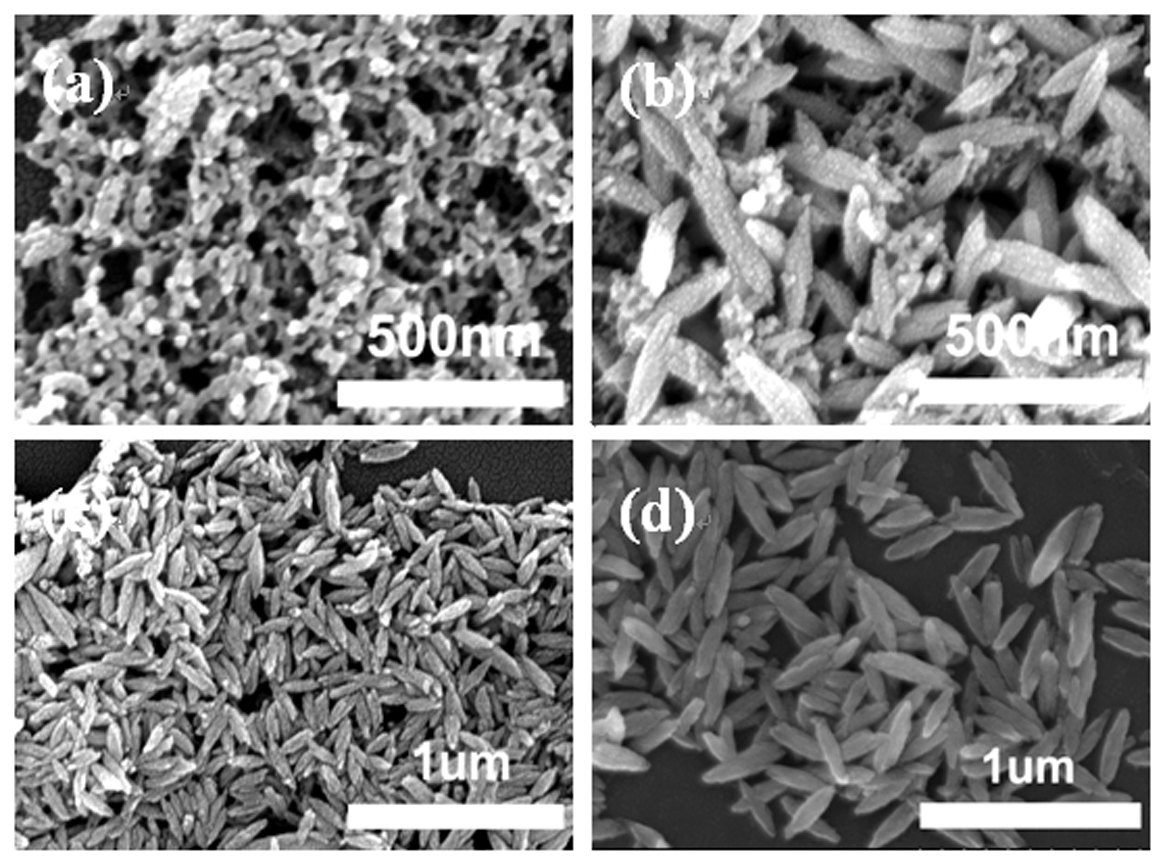


**(c)**

**(d)**

Figure S5. SEM images of the samples obtained over different reaction times with 30 mL ethylene glycol. (a: 0 h; b: 0.5 h; c: 1 h; d: 10 h).

Microflowers were formed after reaction time of 30 min while nanoparticles were observed when 10 mL EG was used. After 1 h, pure microflowers were obtained (Figure S3). Morphology didn’t change even after reaction for 10 h.


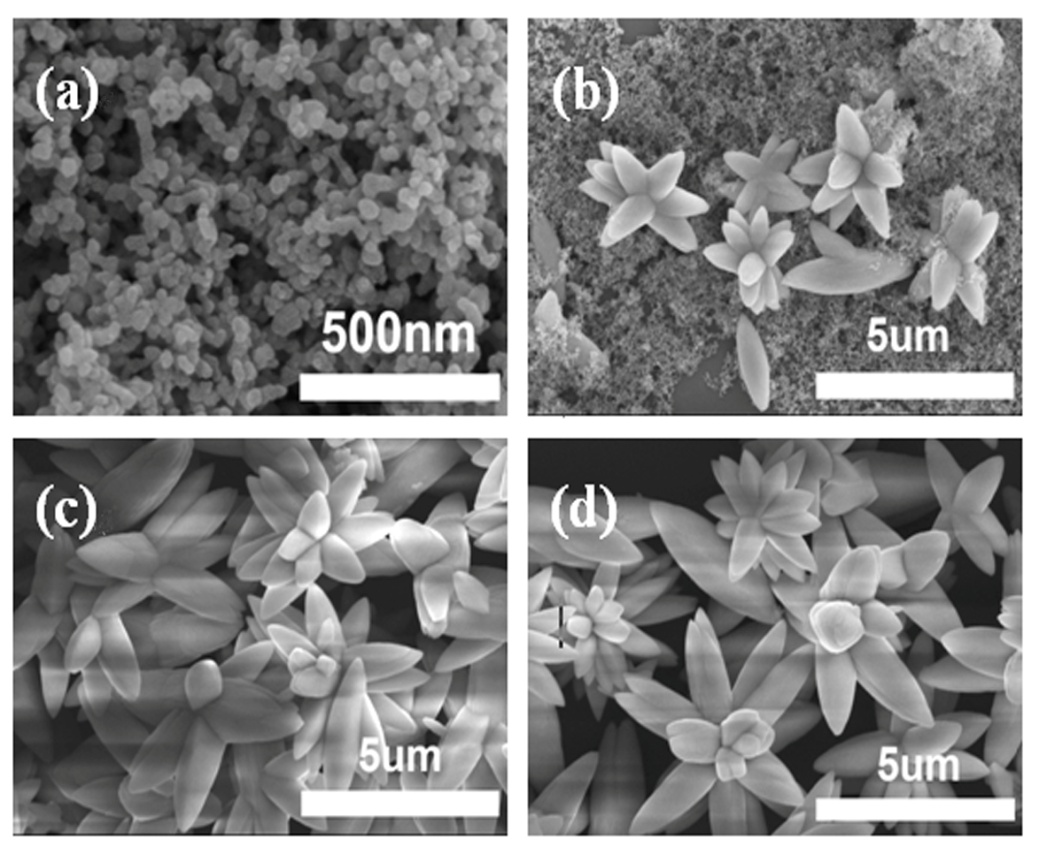


Figure S6. SEM images of the samples obtained over different reaction times with 10 mL ethylene glycol. a, 0 h. b, 0.5 h. c, 1 h. d, 10 h.

Reaction temperature determined the morphology of nanomaterials when the EG amount was remained at the same. Influence of reaction temperatures on the resulting morphologies of nanorods was shown in Figure 2 in details. For the microflower structure, however, the reaction temperature was found to hardly affect its morphology (Figure S4). Just a bit increase in size was observed with increasing reaction temperatures.


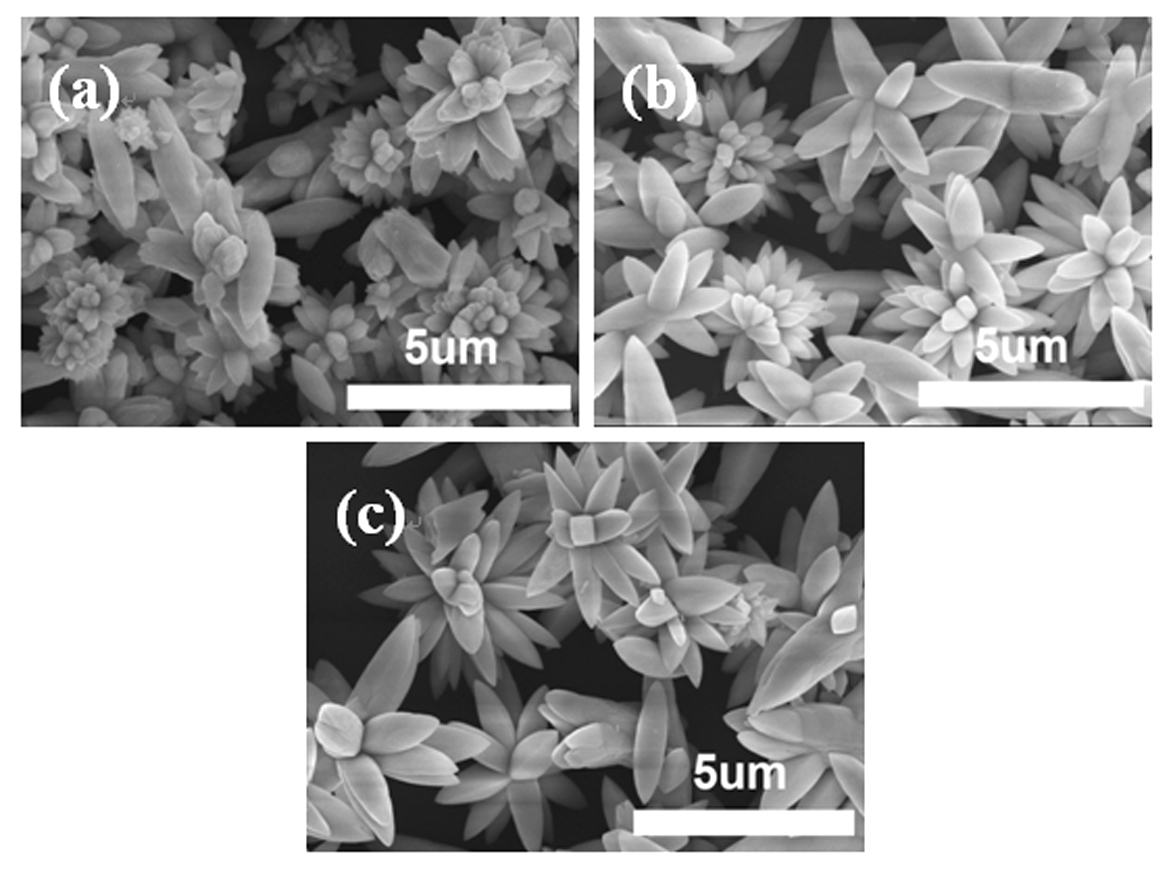


Figure S7. SEM images of the samples obtained at different reaction temperatures with 30 mL ethylene glycol. (a: 30 ^o^C; b: 60 ^o^C; c: 90 ^o^C).
